# Predicting Stimulus Modality and Working Memory Load During Visual- and Audiovisual-Acquired Equivalence Learning

**DOI:** 10.3389/fnhum.2020.569142

**Published:** 2020-10-08

**Authors:** András Puszta, Ákos Pertich, Zsófia Giricz, Diána Nyujtó, Balázs Bodosi, Gabriella Eördegh, Attila Nagy

**Affiliations:** ^1^Department of Neuropsychology, Helgeland Hospital, Mosjøen, Norway; ^2^Department of Psychology, Faculty of Social Sciences, University of Oslo, Oslo, Norway; ^3^Department of Physiology, University of Szeged, Szeged, Hungary; ^4^Faculty of Health Sciences and Social Studies, University of Szeged, Szeged, Hungary

**Keywords:** EEG, working memory load (WML), stimulus modality, machine learing, acquired equivalence associative learning task

## Abstract

Scholars have extensively studied the electroencephalography (EEG) correlates of associative working memory (WM) load. However, the effect of stimulus modality on EEG patterns within this process is less understood. To fill this research gap, the present study re-analyzed EEG datasets recorded during visual and audiovisual equivalence learning tasks from earlier studies. The number of associations required to be maintained (WM load) in WM was increased using the staircase method during the acquisition phase of the tasks. The support vector machine algorithm was employed to predict WM load and stimulus modality using the power, phase connectivity, and cross-frequency coupling (CFC) values obtained during time segments with different WM loads in the visual and audiovisual tasks. A high accuracy (>90%) in predicting stimulus modality based on power spectral density and from the theta–beta CFC was observed. However, accuracy in predicting WM load was higher (≥75% accuracy) than that in predicting stimulus modality (which was at chance level) using theta and alpha phase connectivity. Under low WM load conditions, this connectivity was highest between the frontal and parieto-occipital channels. The results validated our findings from earlier studies that dissociated stimulus modality based on power-spectra and CFC during equivalence learning. Furthermore, the results emphasized the importance of alpha and theta frontoparietal connectivity in WM load.

## Introduction

Working memory (WM) is a cognitive process that enables the retention of information “in the mind” after the physical stimuli that introduced it is no longer available (Atkinson and Shiffrin, 1968; Baddeley, 1992). WM is necessary for “online” information processing, where information storage is obligatory for complex cognitive processes, such as learning, counting, and language acquisition (Baddeley, 2000). The amount of information stored in WM at any given time is referred to as “memory load.”

The prefrontal cortex (PFC; Rypma and D’Esposito, 1999; Haxby et al., 2000; de Fockert et al., 2001) and medial temporal regions (Stern et al., 2001) are primarily involved in WM processes. In general, activities in these areas increase during WM tasks. The ventral PFC is engaged in lower WM load (Paulesu et al., 1993; Awh et al., 1996); however, dorsal PFC activations also occur with increased WM load, typically in a bilateral manner. Moreover, functional neuroimaging studies have revealed that default mode network activity is decreased during WM tasks (Esposito et al., 2009), and such a decrease is correlated with WM load (Medendorp et al., 2011).

Clearly, human scalp EEG oscillatory responses recorded at different frequencies can be related to several aspects of cognitive functioning that range from stimulus processing and attention to WM and long-term memory (Başar et al., 2001; Ward, 2003). For example, increased theta (4–8 Hz) power has been reported to be associated with WM functions (Onton et al., 2005; Sauseng et al., 2010) and WM load (Meltzer et al., 2008). Numerous have demonstrated complex electrophysiological features related to WM tasks. For example, increased theta–gamma cross-frequency coupling (CFC, i.e., the theta rhythm drives the power of gamma oscillations) was described during visual WM tasks (Axmacher et al., 2010; Siebenhühner et al., 2016). Axmacher et al. (2010) clearly illustrated that theta–gamma CFC is highly dependent on WM load.

Increased inter-site phase synchronization (refers to the synchronization of oscillatory phases among various brain regions) has been observed during various memory processes, such as WM maintenance and long-term memory encoding and retrieval (Li et al., 2011). Moreover, studies on phase connectivity have revealed increased gamma phase synchronization during WM (Fell and Axmacher, 2011), whereas others have emphasized the role of alpha and theta band phase synchronizations (Meltzer et al., 2007).

The abovementioned WM tasks were predominantly visually guided n-back tasks (Pesonen et al., 2007), and less information exists on how the abovementioned EEG correlates differ in tasks using different stimulus modalities. Leiberg et al. (2006) emphasized the role of alpha and beta bands in an auditory Sternberg task, implicating that alpha oscillations be associated with the representation of task-relevant stimulus features, while the beta band oscillations could reflect the top-down control of these representations.

At the behavioral level, several studies addressed the effect of different stimulus modalities in WM tasks (for a review, see Quak et al., 2015). The main findings can be summarized in three points. First, the memorizing of modality-specific sensory information is connected to the same brain areas, which were involved in the initial sensory processing (Ranganath et al., 2004; Postle, 2006). Second, the recall of cross-modal objects is more effective than that of the modality-specific objects (Thompson and Paivio, 1994; Goolkasian and Foos, 2005; Delogu et al., 2009). Third, the WM capacity can be higher for cross-modal objects than for unimodal objects (Saults and Cowan, 2007; Fougnie and Marois, 2011).

However, to the best of our knowledge, no study has successfully disentangled EEG parameters connected to WM load and stimulus modality. Thus, the present study aims to analyze how different EEG parameters are primarily involved in predicting WM load and/or stimulus modality using machine learning algorithms.

The EEG datasets previously published by the researchers of the current study and recorded from 18 healthy volunteers during visual and audiovisual associative learning tasks were used (Puszta et al., 2019). The task design was established such that associations were learned using the staircase method, thereby enabling the researchers to investigate the number of associations that participants were required to maintain (WM load) and stimulus modality within the same paradigm.

In the current study, we hypothesized that the accuracy of predicting stimulus modality will be high, at least in terms of prediction based on the power and CFC results, as indicated in the previous publication (Puszta et al., 2019). Instead of using classical statistical methods, the current study applied machine learning classification algorithms to reveal the EEG features considered important for stimulus modality and/or WM load. The use of such classification algorithms has been rapidly increasing, which has been successfully applied to the prediction of different cognitive states as well as clinical disorders with high accuracy (Al-Nafjan et al., 2017). Thus, in the present study, we have focused on what EEG-parameters are important predicting WM-load and/or stimulus modality. How these parameters differ between conditions exceeds the scope of the current study, and has been answered elsewhere (Puszta et al., 2019).

### Materials and Methods

A total of 23 participants were recruited voluntarily from university students (12 females, 11 males, mean age: 26 years, range = 18–32 years). We excluded five participants due to low signal-to-noise ratios. The current study reanalyzed the EEG data of 18 healthy young adults that were previously published (Puszta et al., 2019). The number of samples was identical in both the visual and the audio-visual test. The study protocol conformed to the tenets of the Declaration of Helsinki in all respects and was approved by the Medical Ethics Committee of the University of Szeged, Hungary (Number: 50/2015-SZTE).

#### Visual and Audiovisual Equivalence Test

**Figure 1:**
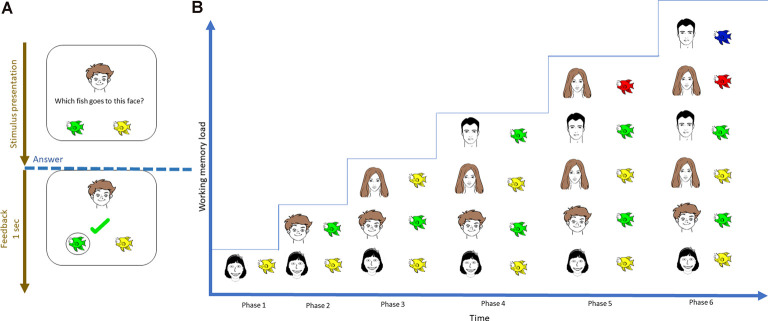
Visual representation of the initial acquisition phase of the visual-acquired equivalence learning paradigm. **(A)** During each trial, the subject was instructed to select one of two possible fish by trial-and-error learning. **(B)** The number of pairs that had to be learned was introduced using the staircase method; therefore, the number of items that needed to be maintained increased with time.

The task design was similar to the previous study [for a detailed description, see Puszta and colleagues (Thompson and Paivio, 1994; Quak et al., 2015) and Eördegh and colleagues (Eördegh et al., 2019; Puszta et al., 2018, 2019)]. Briefly, the visual and audiovisual tests comprised of three phases—acquisition, retrieval, and generalization. During acquisition, the subjects learned visual/audiovisual associations through a trial-and-error mechanism. Each pair was introduced after the subjects correctly answered two times/associations. Hence, the number of associations required to be maintained in WM was increased using the staircase method, where the participants learned six of eight possible pairs. See Figure 1 for a graphical representation of the visual task. During retrieval, the program will ask about the learned pairs. During generalization, the participants were asked about the remaining two associations, which could easily be solved based on the simple rule learned during equivalence learning. The detailed description of both the visual and the audio-visual tasks together with the visual representation of the acquisition phase of the audio-visual task can be found in the Supplementary Material (Supplementary Figure 7; whose basic structure of is the same than that of the visual task, with the sole difference that the associations are built between natural sounds and faces). The present study focused exclusively on the acquisition phase of the visual and audiovisual tasks. The reason we used only the acquisition part of the tasks is that the number of the associations increased only in the acquisition phase but they were constant in the retrieval phases. Thus, only the acquisition phase could shed light on the specific EEG-correlates concerning different levels of WM-load.

The datasets used were published previously in Puszta et al. (2019). The 64-channel EEG datasets (with five-channel EMG) were used during the visual and audiovisual tests recorded using a Biosemi setup. Additionally, 1-min resting state activity was recorded before and after the completion of the visual and audiovisual tests. The number of samples (from the same participants) reached 18 in the visual and audiovisual tasks.

#### Data Analysis

Pre-processing steps were performed within EEGLab based on Makoto’s pre-processing pipeline (Miyakoshi, 2018). EEG data were first high-pass filtered (>0.5 Hz), and noisy channels were interpolated. All trials were visually inspected, and those containing EMG or other artifacts unrelated to blinks were manually removed (on average <6% of trials). Independent components were computed, and components containing blink/oculomotor artifacts or other artifacts that could be clearly distinguished from brain-driven EEG signals were omitted from the data (Miyakoshi, 2018).

The artifact-free EEG datasets pre-processed in EEGLab were then exported to MATLAB 2018b for further analysis.

#### Predicting WM Load and Stimulus Modality From Normalized Power Spectra

**Figure 2:**
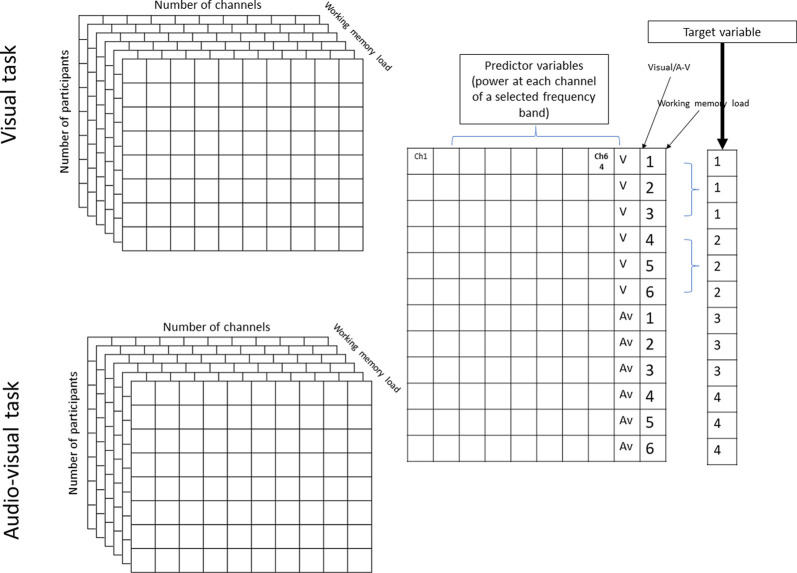
Graphical representation of realignment of population results of one frequency band.

First, the acquisition phase of the EEG data was segmented according to the number of items to be maintained (WM load), thereby resulting in six time-series (Figure 1). The median length of each time series was 28 ± 5 s (range = 21–37 s. Afterward, the power spectrum density of each channel time series was calculated for each segment using a fast Fourier transform. Then, power spectra were normalized with that of baseline activity (1-min segment before and after the task) using decibel normalization (Cohen, 2014). To reduce the number of features during classification, the normalized power spectra of each participant were normalized at conventional frequency bands: θ (4–7 Hz), α (8–12 Hz), β (13–25 Hz), low γ (26–45 Hz), and high γ (55–70 Hz). This step resulted in a 2 × 6 × 64 × 5 matrix for each participant that contained the normalized power values of the two tasks (visual and audiovisual), 64 channels, under six WM load conditions, and five frequency bands, respectively. The matrices were then realigned to train the classification model at each frequency band into a 216 × 64 matrix, where 216 = 18 (number of samples in the visual and audiovisual tasks) × 2 (visual and audiovisual tasks) × 6 (WM load conditions). This matrix was then divided into training cross-validation and a test set using 80-10-10% of the datasets (Moore, 2001). A classification learner app implemented in MATLAB was used to predict the WM load and stimulus modality as a multi-class classification problem, where the following target variables were used: visual task + low WM load (target number 1), visual task + high WM load (target number 2), audiovisual task + low WM load (target number 3), and audiovisual task + high WM load (target number 4). The first three phases of the acquisition part (Figure 1) were labeled as “low WM load, ” and the last three phases as “high WM load.” Figure 2 denotes the visual representation of the matrix realignment.

Previous studies that addressed the performance of different supervised machine learning algorithms on decoding different behavioral states found that support vector machines using Gaussian kernel are probably the best selection (Saputro et al., 2019; Janghel et al., 2020). Therefore, we used the Medium-Gaussian support vector machine algorithm to test the accuracy of prediction at each frequency band. The procedure was repeated 200 times with the dataset randomly divided into the train-cv and test set for each time, thereby obtaining a normal distribution of the accuracy at each frequency band.

As observed in the confusion matrix of the results, the prediction will be higher for target variables 1 + 2 (visual task) and 3 + 4 (audiovisual task), the classification of prediction of stimulus modality was repeated using the same procedure.

#### Predicting WM Load and Stimulus Modality From Phase Connectivity

**Figure 3:**
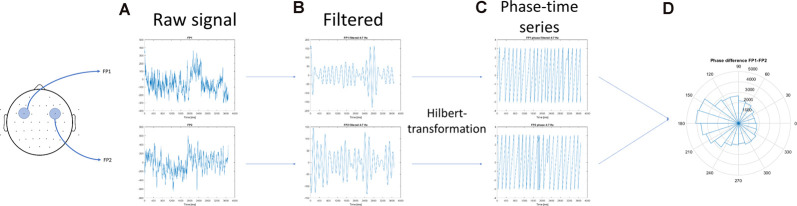
Schematics of the calculation of the inter-site phase coherence. **(A)** The artifact-free electroencephalography (EEG) segments in two (non-neighbor) channels were obtained. **(B)** The concatenated time series were filtered to 5–10 Hz, and **(C)** the Hilbert transformation was performed on the filtered data. **(D)** The mean resultant vector of the phase difference between the two channels was calculated.

In the first step, the acquisition phase of the EEG data was segmented according to the number of items that needed to be maintained (WM load). This separation provided six time-series in each channel according to the six phases of the associative learning tasks (Figure 1). Then, inter-site phase coherence between each pair of non-neighbor channels was calculated in the following frequency bands: θ (4–7 Hz), α (8–12 Hz), β (13–25 Hz), low γ (26–45 Hz), and high γ (55–70 Hz). Phase synchrony analysis was performed as the same procedure suggested by Rodriguez et al. (1999). The steps of individual theta inter-site phase coherence calculations were as follows: (1) raw data of a single participant recorded during the resting state (baseline activity) and the acquisition phase of one task (for example, visual task) were selected; (2) the time series were filtered to 4–8 Hz. The filtering method used a 4 Hz-width, two-way, least-squares FIR procedure (as implemented in the eegfilt.m script included in the EEGLab package; Delorme and Makeig, 2004); (3) the Hilbert transformation was performed on the filtered data; (4) data according to WM load (phases 1–6; Figure 1) and the 1-min baseline activity that preceded the task were segmented, which resulted in seven conditions; and (5) The first and last 200 ms of each condition were omitted to avoid edge artifacts. Figure 3 provides a graphical representation of the above mentioned steps.

Furthermore, steps 1–5 were repeated for another channel, and the mean resultant vector of the phase difference between the two channels was calculated using the Circular Statistic Toolbox (Berens, 2009). To avoid bias in the length of the time series on the resultant vector length, the resultant length [or synchronization index (SI)] was divided by the length of the concatenated time series as follows:

$$\text{SI}={\left|\frac{1}{n}\times \sum_{t=1}^{n}e^{i(\varphi_{ch2,t}-\varphi_{ch1,t})}\right|}^{2},$$

where *i* is the indeterminate unit, *n* denotes the number of time points of the time series, *φ_ch_*_1,*t*_ refers to the phase value of the filtered time series on channel *ch*1 at time point *t*, and *φ_ch_*_2,*t*_ pertains to the phase value of the filtered time series on channel *ch*2 at the same time point *t*. The SI varied between 0 and 1. If the SI was 0, then the phases of the two channels were completely desynchronized. Furthermore, if it was 1, then the phases were completely synchronized.

We then calculated the resultant vector length in each condition and between each possible channel pair (except for neighboring channel pairs) in each subject. Afterward, the phase coherence values were averaged to reduce the number of features in the classification and to compare the connectivity between the four regions of interests (ROIs), namely, left frontal (Fp1, AF7, AF3, F1, F3, F5, F7, FC1, FC3, FC5, and FT7), right frontal (Fp2, F4, F8, FC2, FC6, F2, AF4, FC4, F6, and AF8), left parieto-occipital (P1, P3, P5, P7, CP3, CP5, PO3, PO7, and O1) and right parieto-occipital (P2, P4, P6, P8, CP4, CP6, PO4, PO8, and O2) channels. For example, the phase coherence values of each subject between channels Fp1 and Fp2 were labeled as left frontal–right frontal. This step resulted in a 216 × 64 matrix containing all phase connectivity values of the subjects, where 216 = number of subjects (18) × number of phases of acquisition learning (WM load sections = 6; Figure 1) × 2 (visual and audiovisual tasks) in each frequency band.

This matrix was then randomly divided into training cross-validation and test set using 80-10-10% of the datasets. The classification learner app implemented in MATLAB was used to predict the WM load and stimulus modality as a multi-class classification problem, where the following target variables were used: visual + low WM load, visual + high WM load, audiovisual + low WM load, and audiovisual + high WM load, where the first three phases of the acquisition part were labeled as “low WM load, ” and the last three phases as “high WM load.” The same classification algorithm described in the power-spectrum section was used.

As observed in the confusion matrix of the results that the prediction will be higher for target variables 1 + 3 (low WM load) and 2 + 4 (high WM load), the classification of the prediction of WM load was repeated using the same procedure.

#### Predicting WM Load and Stimulus Modality From CFC

Again, the acquisition phase of the EEG data was first segmented according to the number of items that needed to be maintained (WM load), thereby resulting in six different time series (Figure 1) in each channel. Then, the CFC in each channel was calculated. Based on the previously published results (Puszta et al., 2019), the CFC between the following frequency bands was calculated: θ (4–7 Hz) and α (8–13 Hz) as modulating frequency bands and β (14–30 Hz) and low γ bands (31–45 Hz) as modulated frequency bands. The calculation steps were similar to those suggested by Cohen and those previously published (Cohen, 2008, 2014; Puszta et al., 2019). In the first step, the higher frequency power time series of one channel was extracted from the raw analytical signal. This step was carried out by a combination of band-pass filtering and the Hilbert transformation. First, we have narrow bandpass-filtered the analytic signal to each frequency of the beta and gamma bands (15–45 Hz). The filtering method used a 3 Hz-width, two-way, least-squares FIR procedure (as implemented in the eegfilt.m script included in the EEGlab package). Then, the Hilbert transformation was performed on the narrow bandpass-filtered signal. The power time series was extracted as the squared magnitude of *z*(*t*) and the analytic signal obtained from the Hilbert transformation (power time series: *p*(*t*) = real[*z*(*t*)]2 + imag[*z*(*t*)]2).

Then, the raw analytic signal was band-pass filtered to each frequency of the low-frequency range (4–14 Hz with a 3-Hz-wide FIR filter). The phase of the band-pass filtered low and high-frequency power time series were obtained from the Hilbert transformation of the two time-series.

The synchronization between the phases of the two power time series can be calculated using the SI as follows:

$$\text{SI}={\left|\frac{1}{n}\times \sum_{t=1}^{n}e^{i(\varphi_{lt}-\varphi_{ut})}\right|}^{2},$$

where *n* represents the number of time points, *φut* stands for the phase value of the fluctuations in the high-frequency power time series at time point *t*, and *φlt* denotes the phase value of the low-frequency band time series at the same time point. The SI varied between 0 and 1. If SI is 0, then the phases of the modulating and modulated oscillations are completely desynchronized, whereas if it is 1, then the phases are perfectly synchronized.

In each frequency band comparison (θ–β, θ–γ, α–β, and α–γ), a 216 × 64 matrix was obtained containing the SI value between the two selected frequency bands in each channel, in each subject, and at each WM load segment, for the visual and audiovisual tasks. This matrix was further used in classification analysis.

This matrix was then randomly divided into training cross-validation and test set using 80-10-10% of the datasets. The classification learner app implemented in MATLAB was used to predict the WM load and stimulus modality as a multi-class classification problem, where the following target variables were used: visual + low WM load, visual + high WM load, audiovisual + low WM load, and audiovisual + high WM load, where the first three phases of the acquisition part were labeled as “low WM load,” and the last three phases as “high WM load.” We used the same classification algorithm described in the power spectrum and inter-site phase coherence sections.

Again, as observed in the confusion matrix that the prediction accuracy was higher in stimulus modality cases (Supplementary Figures 1, 2), the classification of the prediction of stimulus modality and WM was we repeated separately using the same procedure.

### Statistical Analysis of Prediction Accuracies

How the prediction accuracies differed from chance level was calculated. The chance level distribution was generated by iteratively (200 times) randomizing the target values of the training test and cross-validation set. To reveal significant differences between predicted accuracies and the abovementioned chance level distribution, analysis of variance (ANOVA) was performed across frequency bands for the modality and/or WM load prediction accuracies. This procedure was repeated for the power spectrum, inter-site phase connectivity, and CFC accuracies. Thus, in the case of the power spectra and connectivity, a 5 × 2 ANOVA was performed (five frequency bands and actual accuracy/chance level). However, in the case of CFC, a 4 × 2 ANOVA was performed (as the number of the modulating–modulated frequency combinations was four).

A significant difference in the prediction accuracies between frequency bands was also verified using one-way ANOVA.

## Results

As all prediction accuracies were significantly different from the chance level, this section reports the results of a one-way ANOVA test, i.e., if the prediction accuracies were significantly different across frequency bands. The Supplementary Material provides detailed results of two-way ANOVA tests (Supplementary Figures 4–6).

### Predicting Modality and WM Load From Power Spectra

**Figure 4:**
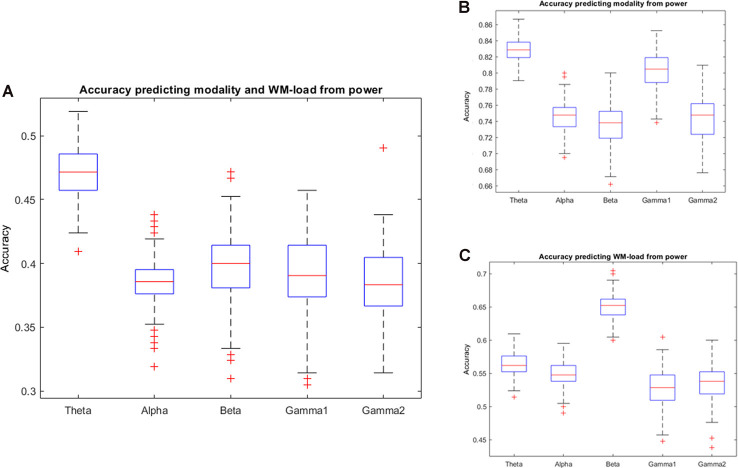
Prediction accuracy results using the power spectra of different frequency bands. Accuracy of results predicting **(A)** Working Memory (WM) load and stimulus modality simultaneously, **(B)** stimulus modality, or **(C)** WM load.

The study found that prediction accuracy was highest in the power spectra of the theta frequency band if the intention is to simultaneously predict stimulus modality and WM load. However, as observed in the confusion matrix of the results, the prediction will be higher for target variables 1 + 2 (visual task) and 3 + 4 (audiovisual task). Thus, the classification of the prediction of stimulus modality was repeated. Indeed, the study found a high prediction accuracy (>80%) for stimulus modality in the theta and low gamma frequency bands. Predicting the WM load from power spectra, the study further found that the prediction accuracy was highest at the beta frequency band. Figure 4 and Tables s 1, 2 provide detailed accuracy results.

**Table 1:** Detailed accuracy results using power spectra.

	Prediction of stimulus modality		Prediction of WM load		Prediction of modality + WM load	
	Mean	SD	Mean	SD	Mean	SD
Theta	0.91	0.02	0.56	0.02	0.47	0.02
Alpha	0.90	0.01	0.55	0.02	0.38	0.02
Beta	0.89	0.02	0.65	0.02	0.40	0.03
Low gamma	0.89	0.01	0.53	0.03	0.40	0.03
High gamma	0.92	0.01	0.53	0.03	0.39	0.03

**Table 2:** One-way ANOVA results of prediction accuracy—differences across frequency bands.

	Modality						
	SS	df	MS	*F*	*p*	Partial eta-squared	Power
Frequency	1.40	4.00	0.35	759.76	<0.01	0.75	1.00
Error	0.46	995.00	0				
WM load
Frequency	1.90	4.00	0.47	998.67	<0.01	0.80	1.00
Error	0.47	995.00	0				
Modality + WM load
Frequency	1.09	4.00	0.27	467.39	<0.01	0.65	1.00
Error	0.58	995.00	0				

*Note: the three table sections contain information about significant differences between frequency bands in predicting stimulus modality, WM load, and both, respectively. SS, sum of square; df, degree of freedom; MS, mean squared; F, F-value; and p, probability*.

### Predicting Modality and WM From Inter-site Phase Coherence Between ROIs

**Figure 5:**
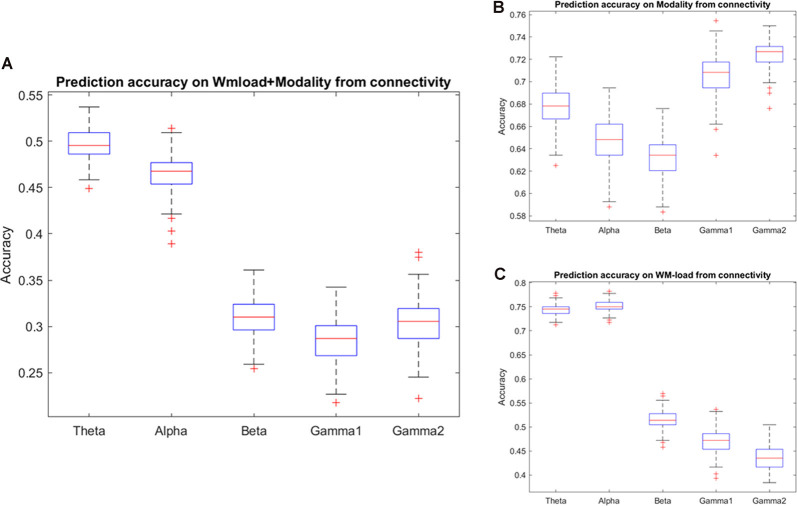
Prediction accuracy results using phase connectivity of different frequency bands. Accuracy results in predicting **(A)** WM load and stimulus modality simultaneously and **(B)** modality or, **(C)** WM load independently.

The study found that accuracy in predicting the WM load and stimulus modality from different frequency band phase connectivities reached chance level except for the theta and alpha bands. As observed in the confusion matrix, the accuracy will be higher for the WM load. Thus, the classification procedure of prediction of the WM load and stimulus modality was repeated from phase connectivity in different frequency bands. Indeed, the study observed that accuracy in alpha and theta phase connectivity was higher than the chance level in predicting WM load. Furthermore, the study found that the prediction accuracy classifying the stimulus modality from high-frequency (beta and gamma) band phase connectivity was highest (~70%). Figure 5 and Tables s 3, 4 illustrate the detailed accuracy descriptions.

**Table 3:** Detailed accuracy results using phase connectivity of different frequency bands.

	Prediction of stimulus modality		Prediction of WM load		Prediction of modality + WM load	
	Mean	SD	Mean	SD	Mean	SD
Theta	0.68	0.02	0.74	0.01	0.50	0.02
Alpha	0.65	0.02	0.75	0.01	0.47	0.02
Beta	0.63	0.02	0.51	0.02	0.31	0.02
Low gamma	0.70	0.02	0.47	0.02	0.28	0.02
High gamma	0.72	0.01	0.44	0.02	0.31	0.02

**Table 4:** One-way ANOVA results of prediction accuracy—differences across frequency bands.

	Modality						
	SS	df	MS	*F*	*p*	Partial eta-squared	Power
Frequency	1.17	4.00	0.29	991.07	<0.01	0.80	1.00
Error	0.29	995.00	0.00				
WM load
Frequency	19.07	4.00	4.77	12998.04	<0.01	0.98	1.00
Error	0.36	995.00	0.00				
Modality + WM load
Frequency	8.42	4.00	2.11	4835.28	<0.01	0.95	1.00
Error	0.43	995.00	0.00				

*Note: the three sections in the table contain information about the significant difference between frequency bands in predicting modality, WM load, and both, respectively. SS, sum of square; df, degree of freedom; MS, mean squared; F, F-value; and p, probability*.

Furthermore, synchronization was highest over the frontal channels in the theta and alpha bands. Supplementary Figure 3 and Supplementary Table 1 present detailed descriptions.

### Predicting Modality and WM From CFC

**Figure 6:**
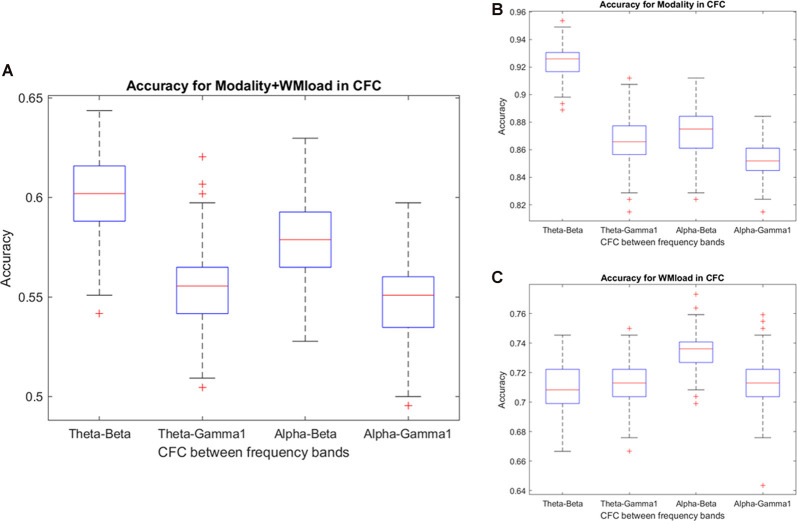
Prediction accuracy using the results of cross-frequency coupling (CFC) between different frequency bands. Accuracy results in predicting **(A)** WM load and stimulus modality simultaneously and **(B)** stimulus modality or, **(C)** WM load.

Notably, no specific frequency constellations in the CFC were observed wherein the prediction accuracy of stimulus modality and WM load significantly differed. However, in the case of stimulus modality, the prediction accuracy was consistently high with the strongest accuracy in the θ–β CFC (>90%). In the case of WM load, no significant difference was found between prediction accuracies. Figure 6 and Tables s 5, 6 present the detailed accuracy descriptions.

**Table 5:** Descriptive statistics of prediction accuracy using the results of cross-frequency coupling (CFC) between different frequency bands.

	Prediction of stimulus modality		Prediction of WM load		Prediction of modality + WM load	
	Mean	SD	Mean	SD	Mean	SD
Theta–beta	0.92	0.01	0.71	0.01	0.60	0.02
Theta–gamma1	0.87	0.02	0.71	0.01	0.55	0.02
Alpha–beta	0.87	0.01	0.73	0.01	0.58	0.02
Alpha–gamma1	0.85	0.02	0.71	0.02	0.55	0.02

**Table 6:** One-way ANOVA results of prediction accuracies—differences across frequency bands.

	Modality						
	SS	df	MS	*F*	*p*	Partial η-squared	Power
Frequency	0.51	3.00	0.17	718.94	<0.01	0.73	1.00
Error	0.19	796.00	0.00				
WM load
Frequency	0.04	3.00	0.01	0.99	0.39	0.00	0.27
Error	20.69	1596.00	0.01				
Modality + WM load
Frequency	0.13	3.00	0.04	1.58	0.19	0.00	0.42
Error	42.54	1596.00	0.03				

*Note: the three sections in the table contain information about significant differences between frequency bands in predicting stimulus modality, WM load, and both, respectively. SS, sum of square; df, degree of freedom; MS, mean squared; F, F-value’ and p, probability*.

## Discussion

The study reanalyzed EEG datasets in a previously published study (Puszta et al., 2019) that were recorded during visual and audiovisual associative learning tasks. Furthermore, the present study investigated the initial acquisition phase of associative learning paradigms. These tasks were established in a manner that provided the opportunity to enable the investigation of different EEG correlates related to WM load and stimulus modality. Several studies have investigated the effect of multimodal integration during WM at the behavioral level (for a review, see Quak et al., 2015). However, less attention has been paid to the effects of multisensory stimuli and probable connected multisensory integration in memory processes. In one of our previous studies, the researchers observed high power alterations and increased θ–β/α–β CFC during audiovisual associative learning tasks compared with the visual task (Puszta et al., 2019). The findings of the present study denoted high prediction accuracy for the prediction of stimulus modality using power spectra and θ–β CFC, thus validating the results from the previous publication. However, θ–β CFC is a seemingly unique feature that may encode differences in stimulus modality. Moreover, CFC between α–β may be influenced by stimulus modality and WM because the current study found that the prediction accuracy for WM load was higher in alpha-beta CFC, whereas it was lower in predicting stimulus modality.

Furthermore, the study observed that prediction accuracy was higher using theta and alpha band phase connectivity. These findings are in line with earlier studies that emphasized the role of theta phase synchronization in memory tasks (Fell and Axmacher, 2011). Several studies on human (Sauseng et al., 2004, 2005) and primate (Taub et al., 2018). EEG have revealed that theta phase synchronization between the PFC and the temporal lobe occurs not only during encoding and retrieval (Sauseng et al., 2004) but also during the maintenance interval of WM (Sarnthein et al., 1998). Theta coupling between parietal and prefrontal cortices is also increased during experimental conditions that require intensive cognitive effort (Sauseng et al., 2005). This tendency indicates that theta coupling may reflect the recruitment of executive control functions. Also, several studies have observed a connection between the amplitude of the theta phase synchronization band and WM load (Payne and Kounios, 2009; Zhang et al., 2016). Previous studies have also demonstrated the relevance of phase synchronization for WM in the beta and gamma frequency ranges. In particular, coherence between the frontal and parietal areas is enhanced in these frequency ranges during the maintenance of information in WM compared with control conditions in humans (Lutzenberger et al., 2002; Babiloni et al., 2004). Intracranial EEG data from patients with epilepsy have revealed a sustained enhancement of beta phase synchronization between extra striatal visual cortical areas and maintenance of complex visual shapes at the same time compared with a purely perceptual control condition (Tallon-Baudry et al., 2001). Furthermore, beta phase synchronization seems to increase between the fusiform face area and medial temporal lobe during high-load WM tasks (Axmacher et al., 2008). The high phase synchronization occurred in cortical areas, which could be responsible for stimulus processing. Thus, the study argues that high-frequency phase synchronization may encode object representations. Taken together, our findings along with earlier studies suggest that low frequency (theta–alpha) phase synchronization is more involved in WM load. However, high-frequency phase synchronization most likely encodes stimulus features, i.e., modality of the stimuli (Fell and Axmacher, 2011).

Publications predicting behavioral data from EEG features (i.e., decoding EEG) using increasingly sophisticated machine learning algorithms have recently been increasing and evolving. As the number of target variables is equal, and the sample size is sufficient compared with the number of features, the researchers are convinced that the high prediction accuracy is representative. As such, the results are in line with earlier suggestions reporting classification accuracy (Billinger et al., 2012).

In summary, we can conclude that power spectra, θ–β CFC, and high-frequency phase coherence are more important features for predicting stimulus modality than for predicting WM load during WM tasks. Furthermore, in line with the findings of earlier studies, the study infers that low-frequency band phase synchronization is a more sensitive EEG feature in predicting WM load than in predicting stimulus modality.

## Data Availability Statement

Publicly available datasets were analyzed in this study. This data can be found here: https://osf.io/5a9yx/.

## Ethics Statement

The studies involving human participants were reviewed and approved by Medical Ethics Committee of the University of Szeged, Hungary (Number: 50/2015-SZTE). The patients/participants provided their written informed consent to participate in this study.

## Author Contributions

APu, BB, GE, and AN designed the study. APu, DN, ZG, and BB performed the assessment and documented the findings. APu analyzed the data. Lastly, APu, ÁPe, and AN organized the study and drafted the manuscript. All authors contributed to the article and approved the submitted version.

## Conflict of Interest

The authors declare that the research was conducted in the absence of any commercial or financial relationships that could be construed as a potential conflict of interest.
