# Supplementary material for: Predicting Stimulus Modality and Working Memory Load During Visual- and Audiovisual-Acquired Equivalence Learning
Source: Front Hum Neurosci. 2020 Oct 8;14:569142. doi: 10.3389/fnhum.2020.569142 (PMC7578848; doi:10.3389/fnhum.2020.569142)
Supplement: Supplementary file 2 [file Data_Sheet_1.docx]

**Supplementary material**

**Description of the two associative learning task**

Visual associative learning test

The testing software (described in earlier studies and originally written for iOS) was adapted to Windows. It was coded in Assembly for Windows and translated into Hungarian, with the written permission of the copyright holder. The paradigm was also slightly modified to reduce the probability of completing its acquisition phase by mere guessing (see below). The tests were run on a PC. The stimuli were displayed on a standard 17- inch CRT monitor (refresh rate 100 Hz) in a quiet room separated by a one-way mirror from the recording room. Participants sat at a 114 cm distance from the monitor. One participant was tested at a time and no time limitation was set. The test was structured as follows: in each trial of the task, the participants saw a face and a pair of fish of different color, and had to learn through trial and error which fish was connected with which face (Fig. [1](https://www-nature-com.ezproxy.uio.no/articles/s41598-019-45978-3#Fig1)). There were four faces (A1, A2, B1, B2) and four possible fish (X1, X2, Y1, Y2), referred to as antecedents and consequents, respectively. In the initial, acquisition stages, the participants were expected to learn that when A1 or A2 appears, the correct answer was to choose fish X1 over fish Y1; given face B1 or B2, the correct answer was to choose fish Y1 over fish X1. If the associations were successfully learned, participants also learned that face A1 and A2 were equivalent with respect to the associated fish (faces B1 and B2 likewise). Next, participants learned a new set of pairs: given face A1, they had to choose fish X2 over Y2, and given face B1, fish Y2 over X2. This was the end of the acquisition phase. To this point, the computer provided feedback about the correctness of the choices, and six of the possible eight fish-face combinations were taught to the participants. In the following phases (retrieval and generalization), no feedback was provided. Beside the already-acquired six pairs (tested in the retrieval phase) the hitherto not shown two pairs were also presented, which were predictable based on the learned rules (tested in the generalization phase). Having learned, that faces A1 and A2 are equivalent, participants were expected to generalize from learning that if A1 goes with X2, A2 also goes with X2; the same holds for B2 (equivalent to B1) and Y2 (equivalent to B1). During the acquisition stages, new associations were introduced one by one, mixed with trials of previously-learned associations. The subjects had to achieve a certain number of consecutive correct answers after the presentation of each new association (4 after the presentation of the first association, and 4, 6, 8, 10, 12 with the introduction of each new association, respectively) to be allowed to proceed. In order to minimize the repetition effect in the acquisition phase, the last, 12-answer trial of the acquisition phase were set to be the part of the retrieval phase. This resulted in an elevated number of the required consecutive correct trials compared to the original paradigm, which made getting through the acquisition phase by mere guessing less probable. Similarly, in the test phase there were 48 trials (12 trials of new and 36 trials of previously-learned associations), as opposed to the 16 trials of the original paradigm.

Audio-visual associative learning test

We developed the Audio-visual (multisensory or bimodal) guided acquired equivalence learning test. The structure of the paradigm was the same as of the visual associative learning test, with the difference that the four antecedents were four sounds (A1, A2, B1, B2) and the consequents were the same four faces as in the visual associative learning paradigm. The main task of the participants was to determine from trial to trial which of the two given faces corresponds to the sound heard at the beginning of the trial. The sounds of the paradigm were a female voice saying “Hello”, the sound of a guitar, the sound of a motorcycle, and the sound of a cat. Each sound lasted less than 1 sec. The category rule implemented in the four faces (i.e. sex, age, hair color) was the same as in the visual paradigm, so similarity across the sounds was not important, but the similarity across the four faces was the same as in case of the visual paradigm. During the acquisition phase, six of the possible eight sound-face combinations were learned. During the test phase, no feedback was provided anymore, but beside the already-acquired six pairs (learned in the retrieval phase), the hitherto not shown last two pairs were also presented (generalization phase).

The description was adopted from Puszta, A., Pertich, Á., Katona, X. *et al.* Power-spectra and cross-frequency coupling changes in visual and Audio-visual acquired equivalence learning. *Sci Rep* **9,**9444 (2019). https://doi-org.ezproxy.uio.no/10.1038/s41598-019-45978-3
