# Supplementary figures and images for: Predicting Stimulus Modality and Working Memory Load During Visual- and Audiovisual-Acquired Equivalence Learning
Source: Front Hum Neurosci. 2020 Oct 8;14:569142. doi: 10.3389/fnhum.2020.569142 (PMC7578848; doi:10.3389/fnhum.2020.569142)

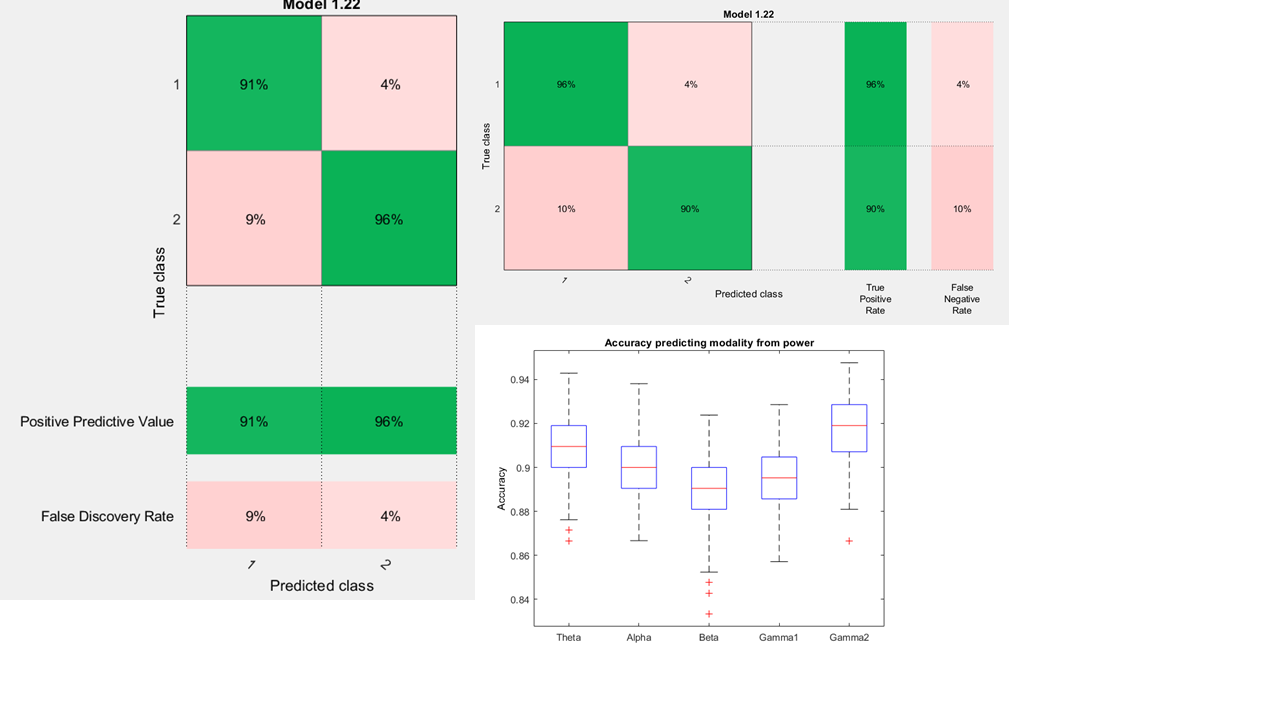

Supplement: Supplementary file 3 [file Image_1.TIF]

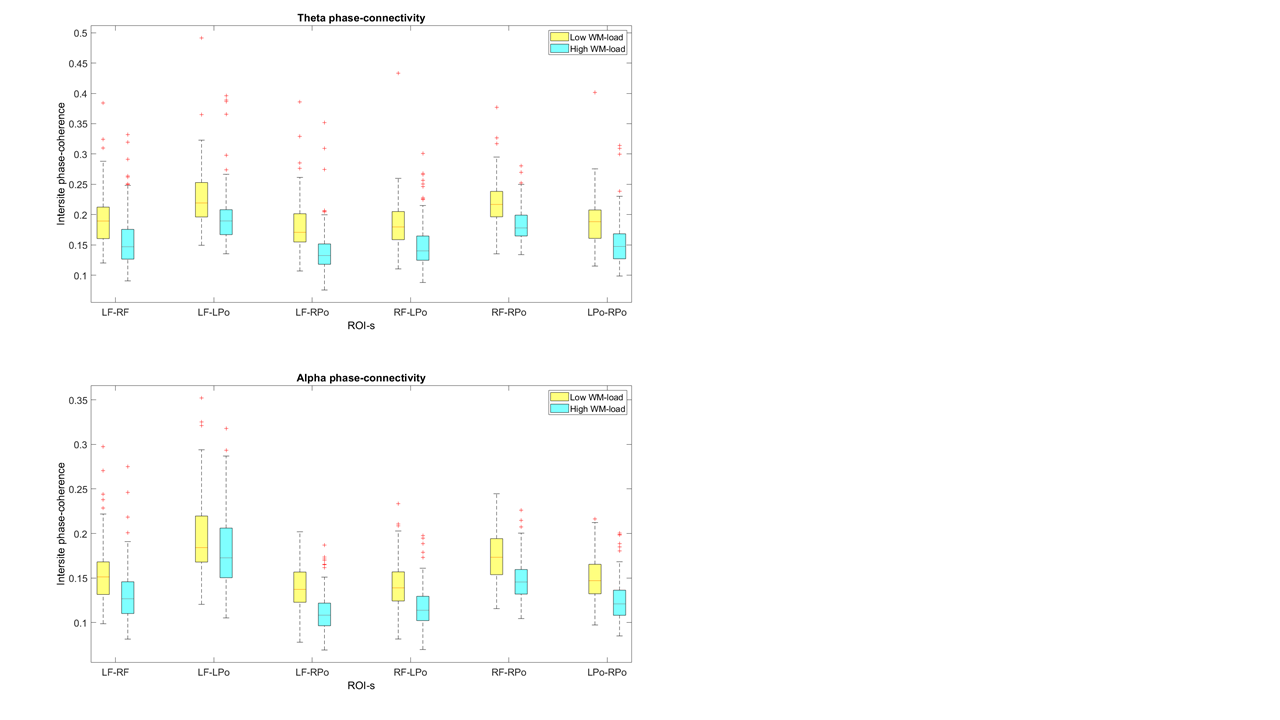

Supplement: Supplementary file 4 [file Image_2.TIF]

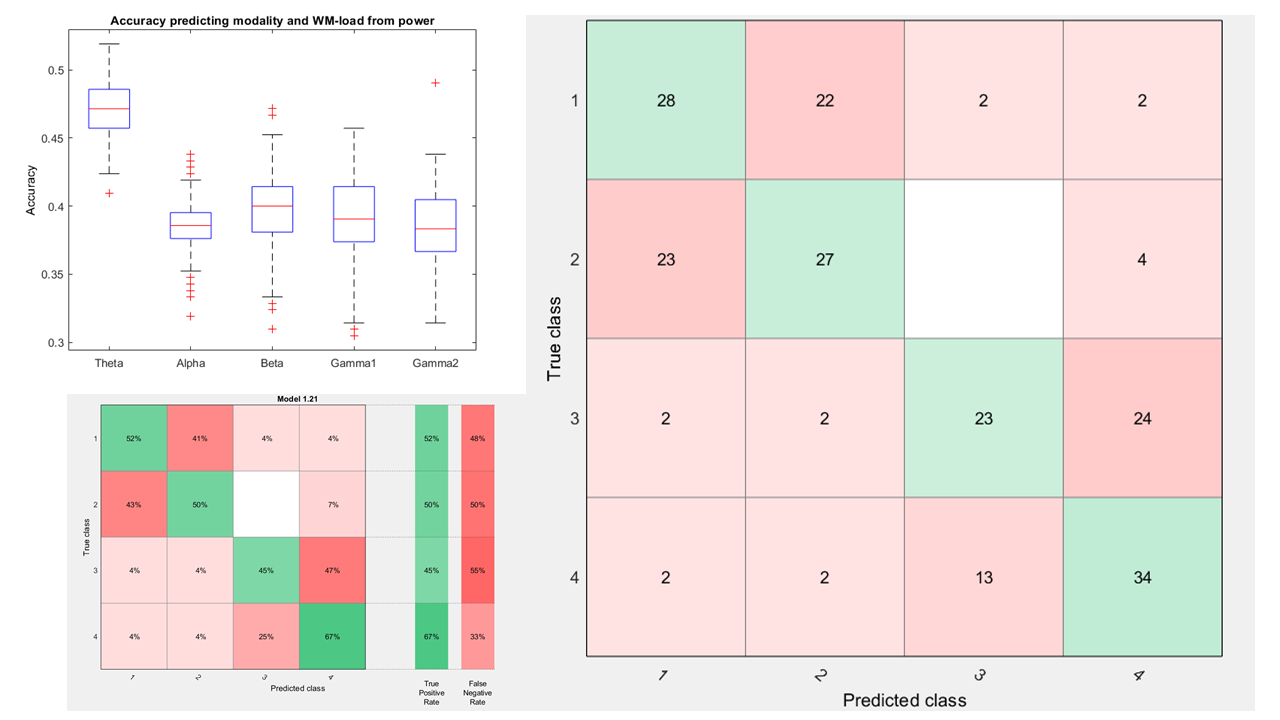

Supplement: Supplementary file 5 [file Image_3.TIF]

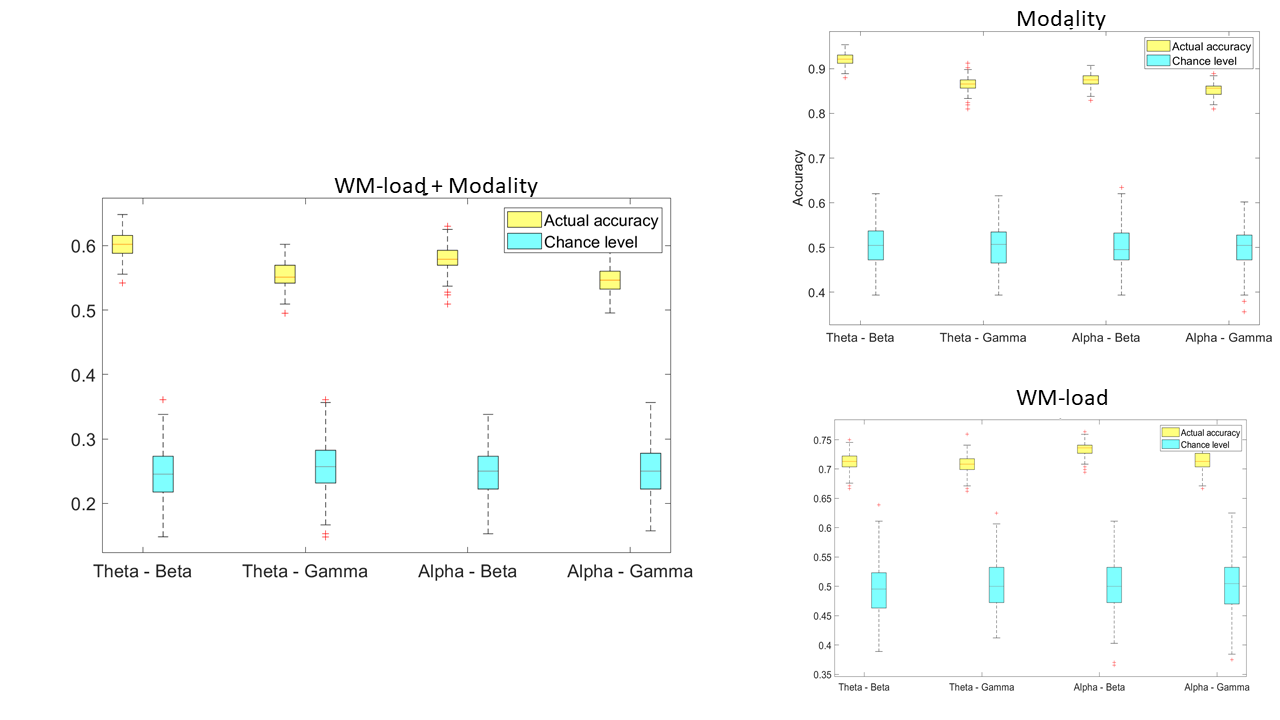

Supplement: Supplementary file 6 [file Image_4.TIF]

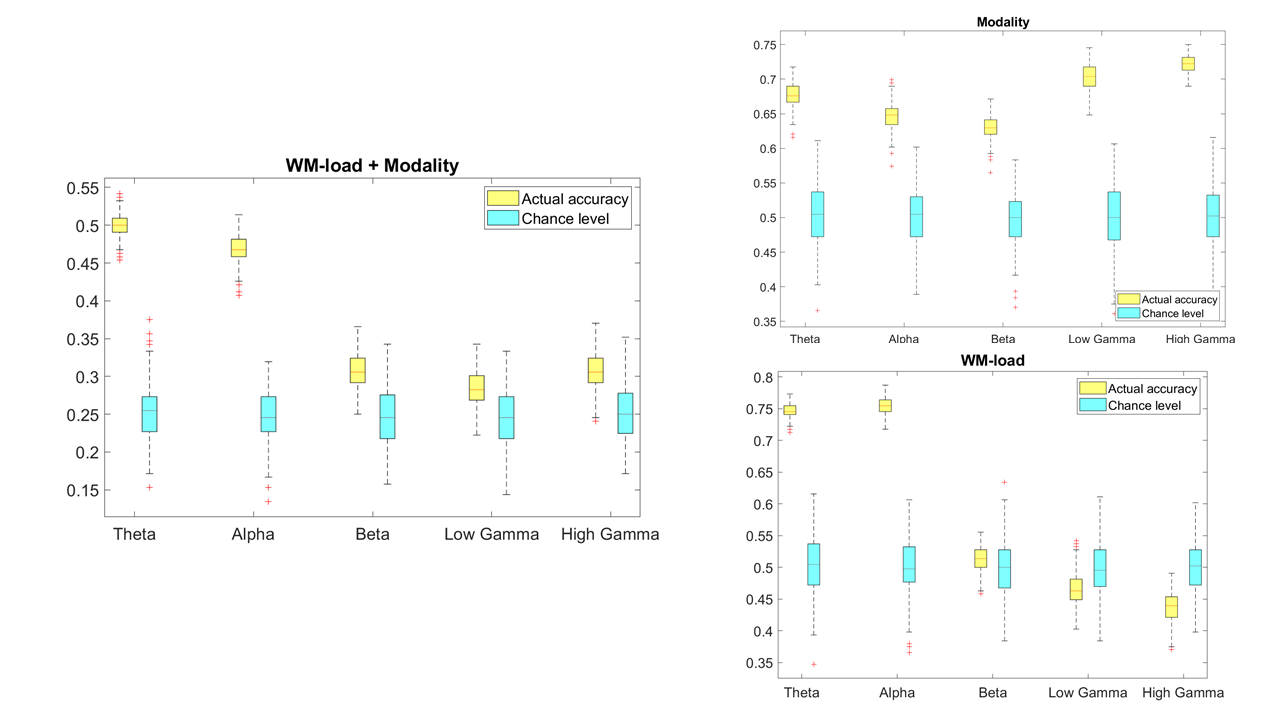

Supplement: Supplementary file 7 [file Image_5.TIF]

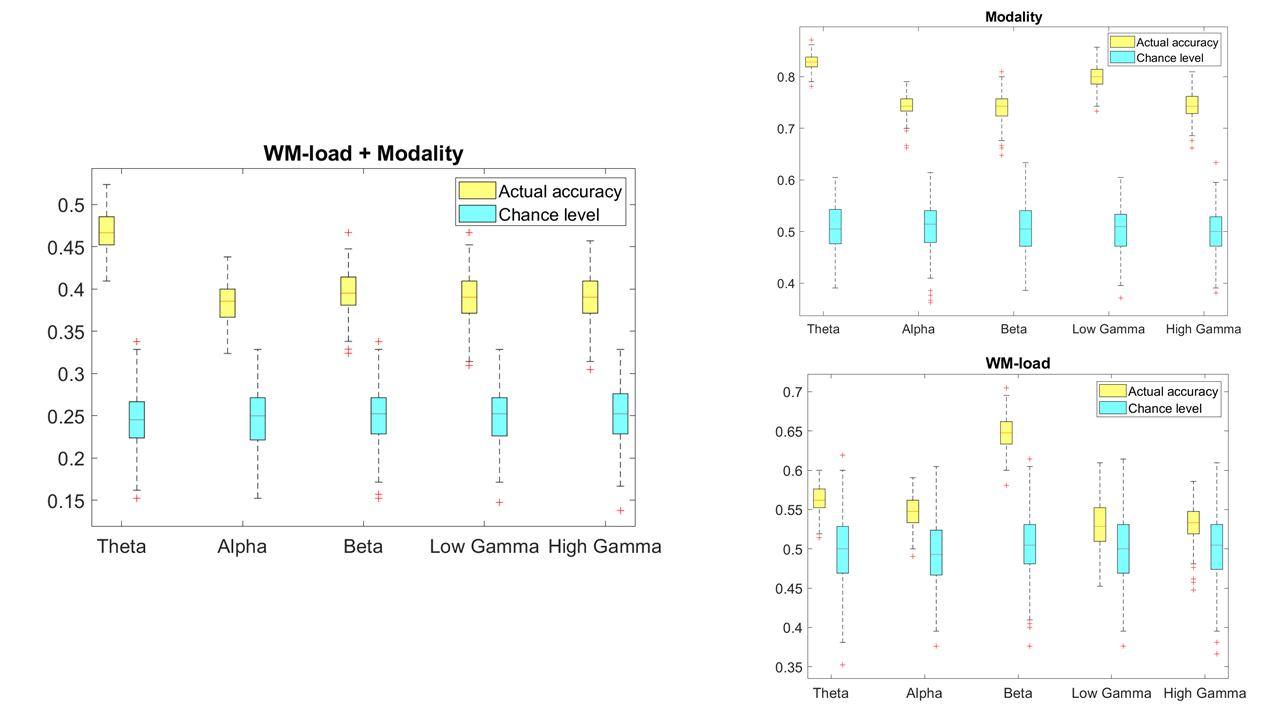

Supplement: Supplementary file 8 [file Image_6.TIF]

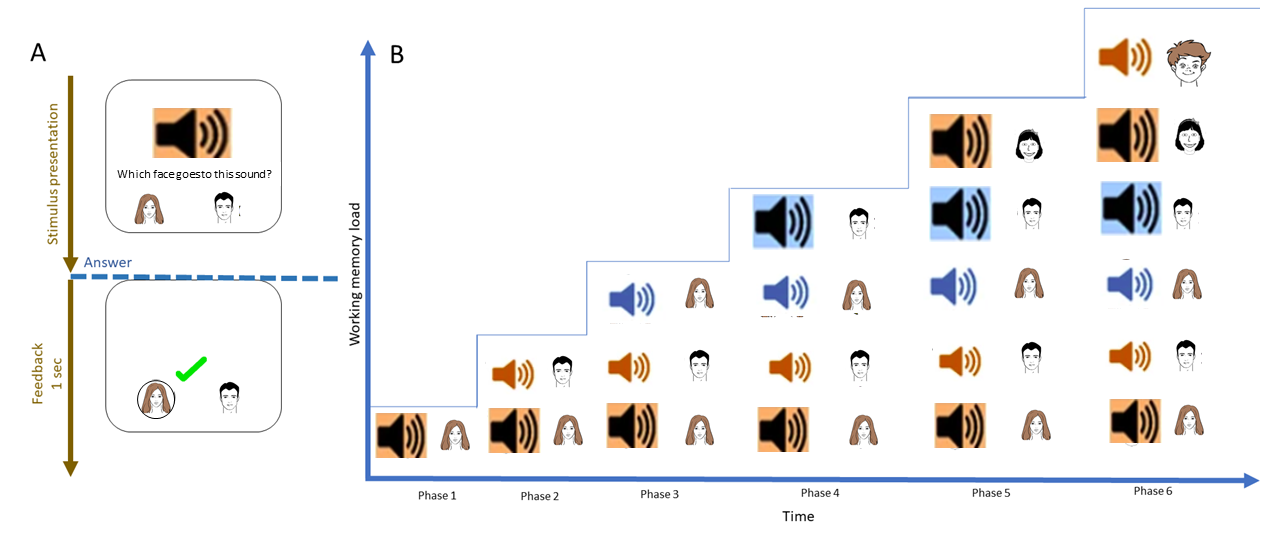

Supplement: Supplementary file 9 [file Image_7.TIF]
